# Two Cases of Myofibrillar Myopathies: Genetic and Quality of Life Study

**DOI:** 10.3390/muscles2020013

**Published:** 2023-04-06

**Authors:** Corrado Angelini, Chiara Ceolin, Alicia Aurora Rodriguez, Vincenzo Nigro

**Affiliations:** 1 Neuromuscular Laboratory, Department of Neurosciences, University of Padova, Campus Biomedico Pietro d’Abano, 35131 Padua, Italy; 2 Geriatrics, University of Padova, 35128 Padua, Italy; chiara.ceolin.1@gmail.com; 3 Neuro-e-Motion Research Team, Faculty of Health Sciences, Department of Psychology, University of Deusto, Av. Universidades, 24, 48007 Bilbao, Spain; aliciarodriguez.b@deusto.es; 4 Telethon Institute of Genetics and Medicine, University of Campania, 80078 Naples, Italy; vincenzo.nigro@unicampania.it

**Keywords:** myofibrillar myopathies, BAG3 mutation, LDB3 gene, socioeconomic impact

## Abstract

We describe two cases of myofibrillar myopathies, due to different gene mutations. The first was a girl with cardiomyopathy and sensory axonal neuropathy that underwent cardiac transplantation at 15 years and suffers from rotatory scoliosis due to BAG3 mutation. The second is a male patient, with evident limb-girdle weakness since age 3. Two muscle biopsies were performed at ages 3 and 15, with muscle MRI, and LDB3 gene sequence analysis also carried out. Muscle biopsies revealed the presence of dystrophic changes in the first biopsy and myopathic abnormalities in the second, and the MRI images of the lower limbs showed an asymmetrical involvement in the thigh of quadriceps muscles and in the calf of gastrocnemius muscles. The patient was responsive to treatment with an intermittent steroid regimen and muscle-strengthening exercises. Considerations on both muscle–bone interaction and psychological and socioeconomic conditions are carried out for both cases.

## 1. Introduction

Myofibrillar myopathies (MFM) are a rather heterogeneous group of neuromuscular disorders caused by mutations of various genes that cause common myopathological features. First of all is the damage of Z-disks followed by myofibrillar disruption and accumulation of multiple proteins in cytoplasmic bodies [1]. Each gene originates from a mutated protein, which is either an integral part of the Z-disk or might be closely associated with it. The expanding panel of disorders associated with myofibrillar myopathies include BAG3, Desmin, Filamin C, Zasp, and all these proteins accumulate in cytoplasmic bodies [2,3,4]. The inheritance pattern of MFM patients follows an autosomal dominant trait, while less frequently these diseases are transmitted by autosomal either recessive or X-linked dominant traits. The clinical signs of various subtypes can be variable and include different ages of onset (from child to adult age) and might involve distal or proximal muscles. Here, we report two cases of childhood-onset MFM resulting from two different gene mutations. For human material, laboratory studies and muscle biopsy consent was given by the Ethical Committee of the University of Padova Hospital on 1 March 1995 and the investigations were carried out, following the rules of the Declaration of Helsinki of 1975. Each participant signed an informed consent form and gave informed consent to the utilization of pictures for scientific purposes.

## 2. Case 1. Childhood Case of a Thin Girl with Multiple Contractions, Scoliosis and Rigid Spine

### 2.1. History

**Figure 1:**
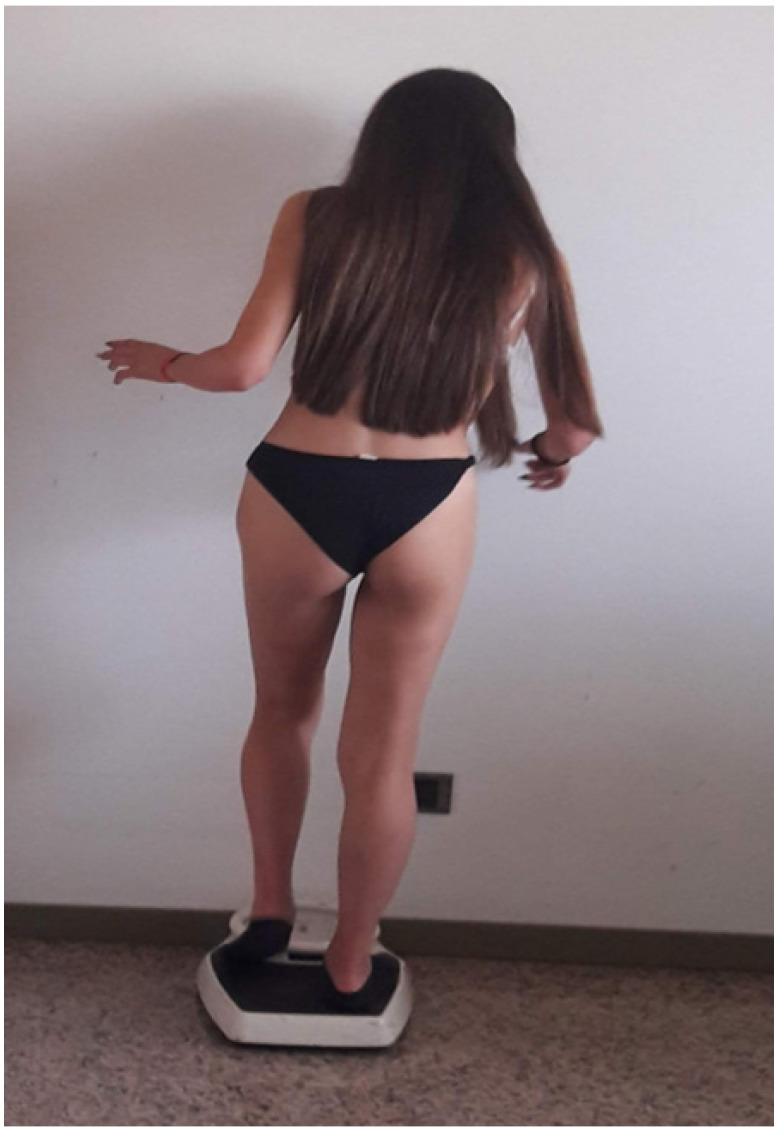
Case 1 is a thin girl with multiple contractures, scoliosis, and a rigid spine. She had both distal and proximal weakness, and at 14 years she had gait disturbance and thin legs for restrictive-hypertrophic cardiopathy, for which she underwent transplantation.

MFM with cardiac transplant due to BAG3 myopathy in an 18-year-old girl. This case was followed from 3 to 18 years for myopathy, heart involvement, pancreatic tumor after the heart transplant, and rotatory scoliosis (Figure 1). She was a Caucasian woman with a weight of 46 kg, a height of 162 cm and was 18 years old.

A thin girl in her early teens presented restrictive cardiomyopathy and underwent a heart transplant at 14 years of age. The transplantation caused some respiratory difficulty, and later she had severe diffuse muscle atrophy and weakness, presented contractures at the knees and ankles, had footdrop, and had an increased serum creatine kinase (CK) level.

This girl manifested exercise intolerance from age 3 and by cardiac ECHO was found to have restrictive cardiomyopathy and tele-diastolic reflow. At age 11, a mutation for a heterozygous variant (p.Pro290Leu) was found and she was diagnosed with myopathy due to BAG3 gene. This sequence change replaces proline with leucine at codon 290 of the TREX1 protein. She had hypertrophic cardiomyopathy and peripheral neuropathy. In a neurological exam, she walked with a waddling gait, presented bilateral winging scapulae, raised with two hands on the floor in Gowers’ maneuver, and had distal wasting, weakness and erythromelalgia. Due to shortness of breath, she was treated with bisoprolol 1.25 mg/day and aldactazide 25 + 25 mg (hydrochlorotiazide + spironolactone) and slept with her head on one cushion. During cardiac catheterization, Case 1 had episodes of ventricular systolic hypertension. A cardiac biopsy was performed, during catheterization, which showed lymphocytic infiltrates and diffuse fibrosis. At age 12, an open quadriceps muscle biopsy was performed and the muscle was frozen and stained according to Dubowitz histochemical techniques [5], showing numerous splitting fibers, internal nuclei, cytoplasmic bodies, and rimmed vacuoles. At age 14, she underwent a cardiac transplant, was put in the Intensive Care Unit, and presented nocturnal episodes of apnea. Her scoliosis deteriorated, and she had to undergo immunosuppression. Slowly, her motor function improved.

### 2.2. Diagnosis

A muscle MRI revealed a fibro-fatty substitution of the scapular girdle more pronounced on the right side, bilaterally in quadriceps thigh muscles, and present in gastrocnemius muscles. At age 16, she was found to carry a pancreatic tumoral cyst that was resolved. Quality of life (QoL) was investigated by a quality of life questionnaire for neuromuscular disease (INQoL), an instrument created to measure QoL in patients with neuromuscular diseases [6]. The results revealed a significant impact on her daily life as a result of muscle weakness, fatigue, and muscle pain, as well as a decrease in her independence as a consequence of her illness.

The whole picture was particularly severe, but it was found that her QoL was not particularly affected by DEXA scan osteopenia. Scoliosis has been followed, but a surgical correction is not indicated.

This girl with MFM due to BAG3 mutation has been given a heart transplant, likely due to immunosuppressive treatment she developed a pancreatic tumor, and the main complaint currently is the bone complication, as shown in the picture.

The mutation affects p.Pro209Leu might alter the folding of BAG3, as demonstrated by Selcen et al. [3]. Additionally, it might affect allosterically the binding properties of the classical BAG3 domains. Our clinical findings indicate new clinical features of such muscular dystrophy, i.e., sensory neuropathy, rotatory scoliosis and cardiomyopathy, and as a muscle disease caused by a spontaneous variant in BAG3 family of proteins, with both scoliosis and osteopenia.

## 3. Case 2: An Asymmetric Limb-Girdle Myopathy Associated with the LDB3 Variant, Is Found in the Propositus (Figure 2)

### 3.1. History

LIM domain binding 3 (LDB3) gene domain is a gene that can give different clinical phenotypes. In a family, we studied a 37-year-old man with upper asymmetrical grip strength, high CK, and limb weakness that presented steroid responsiveness and had a known variant in the LDB3 gene.

**Figure 2:**
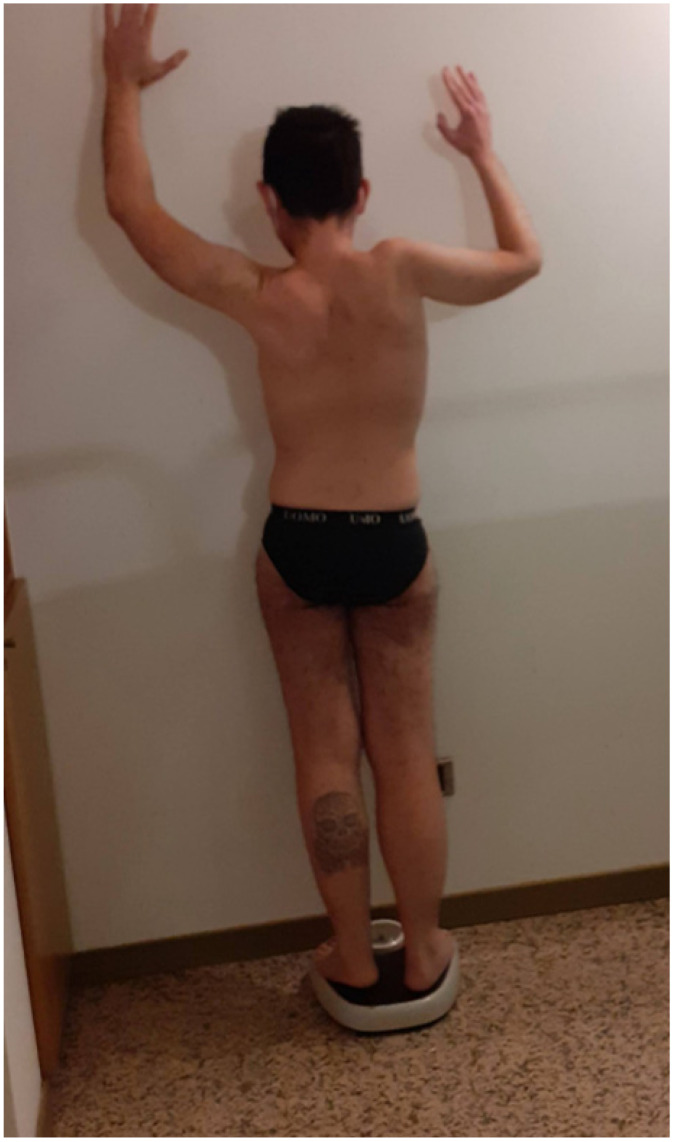
Case 2: The index case had a phenotype consistent with limb-girdle myopathy and scoliosis, asymmetric features in the upper extremities on the right.

The mother had 10 abortions. Weight: 40 kg. Height: 160 cm. Age: 70 years. She had three children, one premature baby died, a daughter 38 years, the proband 37, and one female daughter 34 years, she had neck weakness muscle weakness, atrophic tongue, and was depressed for the death of the husband. The father died of a lung tumor. There was familiarity on the father’s side for ischemic cardiopathy.

The proband was a Caucasian man with a height of 162 cm, and a weight of 48 kg. This child at 3 years of age had numerous falls, difficulty climbing stairs, waddling gait, raised creatine phosphokinase (CPK), and was suspected to have muscular dystrophy. An open quadriceps muscle biopsy was performed on the child and there were myopathic features, with several atrophic fibers, at immunohistochemistry, faintly staining for dystrophin, and positive for acid phosphatase. A provisional diagnosis of an intermediate case of dystrophinopathy was carried out. At 15 years, an open biceps biopsy was repeated and only slight myopathic signs were found. Calpain, dystrophin, and alfa sarcoglycan were found normal and a provisional diagnosis of limb girdle muscular dystrophy (LGMD) was entertained. On ECHO cardiac exam he had a systolic function borderline low and a diagnosis of cardiomyopathy, and a class I New York heart association scale (NYHA) was performed.

**Figure 3:**
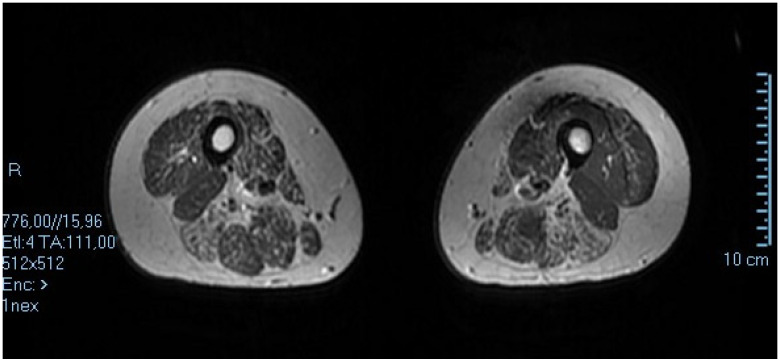
Muscle MRI of proband’s thigh at age 35 shows asymmetric involvement of both anterior vastus lateralis and posterior semitendinosus and semimembranosus muscles with evidence of connective tissue replacement.

At age 35 a muscle MRI was performed (Figure 3) with a 1.5 Tesla Siemens Apparatus and analysis of DNA showed a normal Fukutin related protein (FRKP) gene, but a variant was traced in the LDB3 gene. Mutation in the LDB3 gene can be associated with cardiomyopathy, dilated, with or without left ventricular noncompaction myopathy, myofibrillar myopathy, and familial isolated dilated cardiomyopathy, and late-onset distal myopathy.

### 3.2. Genetic and Psychosocial Assesment

DNA was obtained by the whole family and fully investigated at TIGEM. A Next Generation Sequencing test was carried out of the exonic sequences of 2742 genes associated with known pathologies and enriched with Sure Select technology, Agilent (Inherited Disease Panel) and a diagnostic confirmation by means of bidirectional capillary sequencing was obtained. A dual energy X-ray absorptiometry (DEXA) scan was performed in the index case and revealed osteopenia. Muscle MRI showed asymmetric quadriceps muscle involvement and gastrocnemius muscles of the calf, and grip strength was evaluated with a hand myometer resulting as much lower on the right side.

We found an LDB3 variant in the index case the mother and the sister. The variant was on gene LDB3, chromosome 10, exon 10, c.1487 C > T, Phe496Ser.

The index case had a phenotype consistent with limb-girdle myopathy and scoliosis, but neurogenic features in the upper extremities on electromyographic exam (EMG). The patient was extensively studied by muscle biopsy, muscle MRI, grip strength, and DEXA scan, consistent with bone osteopenia. The muscle biopsy showed dystrophic features, normal dystrophin, calpain, sarcoglycans, and dysferlin. In DNA, a variant in LDB3 was found in three members of the family, and a VUS in plectin and SGCA genes in the index case. MR imaging of lower limbs showed asymmetrical involvement both of the quadriceps muscles of the thigh and gastrocnemius muscles of the calf.

In this case, two instruments were used to measure QoL and the economic impact that this disease had on the patient. According to his responses, the highest expenses were for private doctors (1000–2000 euros per month), physiotherapy (2000–3000 euros per month), external caregivers (1000–2000 euros per month), and attendance at conferences on their illness (100–500 euros per month). On the other hand, responses on INQoL revealed that symptoms of muscle weakness, locking, and fatigue had a severe impact on their daily life.

### 3.3. Treatment

Regarding treatment, the patient has undergone intermittent deflazacort therapy (30 mg/kg/alternate day), as in DMD [7] with benefit and performed four times a week muscle-strengthening activity.

What defines a muscle-strengthening activity is that, in general, a muscle-strengthening exercise, performed as strength/weight/resistance exercise or training, is a series of voluntary activities that includes the use of either exercise bands or weight machines, or their own body weight, including push-ups or sit-ups: a definite benefit was referred by this patient on this type of physical therapy treatment, performed for one hour 4 times weekly.

## 4. Discussion

The girl with MFM due to BAG3 mutation has been given a heart transplant, due to immunosuppressive treatment she developed a pancreatic tumor, and the main complaint currently is bone complications, as shown in the picture. We hypothesize that p.Pro209Leu may alter the folding of BAG3; alternatively, it may allosterically affect the binding properties of the classical BAG3 domains [8]. The Pro209Leu variant might affect the molecular property of the wild-type protein, as this is the most common mutation of cases described in Table 1, but our findings define new clinical features in this childhood onset myopathy and cardiomyopathy, and as a disease characterized by a spontaneous mutation in the BAG3, with neuropathy, rotatory scoliosis and osteopenia.

### 4.1. Genetic and Clinical Approach

MFMs represent a genetically heterogeneous subset of muscular dystrophies, characterized by a morphologically distinct trait. Cardiomyopathy, peripheral neuropathy, and sporadic or dominant inheritance are features that are frequently associated [1,2,3,4,5,7,8,9,10,11,12,13,14]. The histological change is peculiar since it is produced by disintegration of the myofiber Z disk as an early pathological alteration; this is followed by damage of the myofibrils, follows the aggregation of degraded filaments in granular or hyaline inclusions, and produces an intracytoplasmic expression of multiple Z disk-related proteins in cytoplasmic bodies. We here present the cause of MFMs in the first of the two unrelated patients in the first due to a missense mutation in the BAG3 protein encoded by BAG3, and in the second case to a variant of the LDB3 gene [15]. BAG3 is an anti-apoptotic protein that localizes near the Z disk. In Patient 1, ultrastructural observation of nuclear apoptosis was an additional clue pointing to BAG3 as the responsible gene. The rigid spine of Patient 1 was of special interest. This clinical feature is also often present in other myopathy patients such as those with selenoprotein N1 mutations with features of Mallory body-like inclusions, in minicore disease, in the congenital fiber type disproportion, and is frequently observed in variants in FHL1 associated with cytoplasmic inclusions that reduce NBT and thus stain strongly with the Menadione-NBT. Rigid spine can also be seen in other myopathies due to variants in collagen 6, lamin A/C. and emerin, and less frequently in other muscle diseases, such as dysferlinopathy, and this pattern suggests a link between muscle weakness and bone abnormality. Our first patient had the same c.626C > T mutation, common in most other BAG3opathy cases. She presented clinical signs in early childhood followed by severe lower limb weakness, especially distal muscles with MRC grade 3/5.

Ten years into disease progression, the patient presented respiratory failure caused by cardiac transplantation. Since then, she had been intermittently using a non-invasive ventilator (BiPAP) for 5–7 h per night for 6 days per week, as planned by a local pneumologist. The patients had axonal sensorimotor polyneuropathy confirmed by EMG.

In Patient 2, a ZASP variant was found in the proband, his mother, and his sister. Since in this second case we found an LDB3 variant in the index case, but also in his mother and the sister, its role has to be ascertained, since only the proband and mother reported clinical signs: while the proband had a long-lasting weakness of the upper and lower girdle with asymmetry in grip strength, the mother presented an axial weakness of the neck muscles and arthritis responsive to therapy. In this case, we carried out two muscle biopsies, muscle MRI (Figure 3) and LDB3 gene sequence analysis. The first muscle biopsy was indicative of changes compatible with the dystrophic process, the second one presented myogenic changes in the proband at age 15, and the subsequent MRI imaging studies in the lower limbs showed asymmetrical involvement of thigh quadriceps muscles semitendinosus and semimembranosus and calf gastrocnemius muscles.

The variant was on gene LDB3, chromosome 10, exon 10, c.1487 C > T, Phe496Ser.

The index case had a phenotype consistent with limb-girdle myopathy and scoliosis, but neurogenic features in the upper extremity. The present family presents the LDB3 variant, but only the index case had other genetic variants; therefore, the whole genetic load has to be considered to understand the impact on the clinical phenotype since the ZASP variant was found in the proband, his mother, and his sister.

PDZ motif-containing protein-related myofibrillar myopathy Z disc-associated, alternatively spliced (ZASP-MFM) is a rare autosomal dominant or late-onset distal myopathy with related cardiomyopathy, which is produced by mutations of LIM domain-binding 3/Z band alternatively. We here report the clinical, myopathological and genetic findings, and muscle MRI changes in an Italian patient. Two muscle biopsies, muscle MRI, and LDB3 gene sequence analysis were carried out in the index case. Muscle biopsies showed the presence of muscular dystrophy compatible changes at 3 years and myogenic features at 15 years in the proband, and the MR images of the lower limbs showed the rather asymmetrical involvement of thigh quadriceps and posterior muscles and calf gastrocnemius muscles. Gene analysis for LDB3 revealed an heterozygous variant in exon 10, c.1487 C > T, Phe496Ser in the pedigree, similar to what was recently described in an Asian case [16] (Table 2). To explain the different phenotypes, one has to acknowledge the possibility of an additive effect in the proband of additional variants found in alfa sarcoglycan and plectin genes. These missense mutations, as with several mutations localized in the extracellular domain of alpha-sarcoglycan, might result in the change of a positively charged amino acid. Our results suggest that patients with partial alpha-sarcoglycan or dystrophin deficiency exhibit this as a secondary consequence of other genetic disorders. In fact, we report here two patients’ disorders linked to Z disk, the second with low immunohistochemical stain for dystrophin. Altogether, the dystrophin laminin complex represents a major axis from the Z disk to extracellular matrix: there is an expanding series of genes associated with MFM along this axis, i.e., DES, CRYAB, MYOT, LDB3, FLNC, BAG3, and FHL1 [6,17,18,19,20].

### 4.2. Therapeutic Approach

The first case presented with severe cardiomyopathy and the main therapy was represented first by cardioactive drugs, followed by cardiac transplantation with some complications since she had a weak, fragile muscle build. Another complication was the occurrence of a pancreatic tumor, probably related to the heavy immunosuppression for the heart transplantation, an unresolved problem is still the rotatory scoliosis, and the psychological pattern related to her body image. Although QoL is not particularly deteriorated, she still needs psychological support.

### 4.3. Psychosocial Issues

The second case, although permanently affected, has a normal life and a family, and he was able to cope with his myopathy. On the other hand, although it was not possible to assess the economic impact of one of the cases, it shows that there is a substantial economic burden, which is assumed by the patients in the absence of support or subsidies. It has been shown that this burden, in the long term, can have a serious impact on their physical and mental health [21,22], and it is therefore recommended to pay special attention to these aspects. In terms of QoL, the results revealed in both cases that QoL was affected by muscle weakness and fatigue. The impact of fatigue caused by the disease was more severe in these two cases than in other neuromuscular pathologies [23,24]. QoL has not been sufficiently investigated in these conditions, and it is relevant to explore it to know the aspects of life that are most affected specifically by these diseases. In this way, treatments can be directed at improving the overall QoL of the patient.

### 4.4. Physical Therapy

Part of the physical treatment Case 2 is doing is a muscle strengthening activity, therefore such activity appears to be indicated, rather than contra-indicated in this case of myopathy. The body’s bones and muscles respond to weight-bearing activities and work against the forces of gravity since, while the patient is standing, the skeletal muscles pull on the bones. This type of action and normal walking increases or preserves bone density.

A different consideration regards weight-bearing exercise that can be of high- or low-impact and there is controversy on their beneficial role in myopathy cases. We recommend low-impact weight-bearing exercises as possible exercises for patients, both for balance issues or to prevent risks. Activities that incorporate some form of resistance, usually weights, against the body are strength-training exercises, which are recommended. By putting moderate stress on the muscles and bones, strength-training exercises might help preserve muscular strength and improve bone density

The main aim in myopathy performing the strength-training exercise is to maintain muscle mass to improve patient balance and strength, which might help to decrease risk factors for falls and bone fractures. While there are some benefits to low grade exercise (such as yoga), it is important to observe that some positions might be inappropriate for muscle patients who have osteoporosis or are at increased risk of fractures, but substitutions or exercise modifications that carry similar effects might be performed. The viable relation has different features according to age and gender, and demonstrates that both tissues in real life and in several myopathic disorders, including MFM, have interdependence and should be considered together during rehabilitation.

## Figures and Tables

**Table 1:** Clinical signs in patients with BAG3 myopathy.

Reference	Age Atonset (Years)	Features Atonset	Cardiomyopathy	Contractures	Weakness	Peripheral Neuropathy	Respiratory Failure
Odgerel et al., 2010 [2]	5	Not reported	Restrictive- hypertrophic	Not reported	Yes	Axonal neuropathy	Yes
Odgerel et al., 2010 [2]	12	Not reported	Restrictive- hypertrophic heart transplantation	Not reported	Yes	Axonal neuropathy	Yes
Odgerel et al., 2010 [2]	12	Pes cavus, weakness, cardiopathy	Restrictive- hypertrophic	Scoliosis and rigid spine	Distal weakness and neck weakness	Axonal neuropathy	Yes
Odgerel et al., 2010 [2]	5	Gait disturbance	Restrictive- hypertrophic heart transplantation	Not reported	Proximal weakness	Axonal neuropathy	Yes
Lee HC et al., 2012 [8]	6	Gait disturbance	Restrictive- hypertrophic	Multiple contractures and rigid spine	Mild proximal weakness	Axonal neuropathy	Not reported
D’avila et al., 2016 [4]	11	Contractures	Hypertrophic and arrhythmias	Rigid spine	Proximal weakness	Axonal neuropathy	Yes
Selcen et al., 2009 [3]	Toddler	Toe walker	restrictive heart transplant	Toe walker	Severe weakness	Not reported	Yes
Selcen et al., 2009 [3]	13	Scoliosis rigid spine, fatigability	hypertrophic	Scoliosis and rigid spine	Distal and proximal weakness	Axonal demyelinating neuropathy	Yes
Selcen et al., 2009 [3]	Toddler	Toe walker	Restrictive	Scoliosis, rigid spine and toe walker	Progressive proximal weakness	Not reported	Yes
Jaffer et al., 2012 [9]	Toddler	Toe walker	Restrictive-heart transplantation	Multiple contractures and rigid spine	Distal and proximal weakness	Axonal neuropathy	Not reported
Jaffer et al., 2012 [9]	Toddler	Toe walker	Restrictive	Multiple contractures, scoliosis and rigid spine	Distal and proximal weakness	Axonal neuropathy	Yes
Kostera Pruszczyk et al., 2015 [10] Present Case	12 3	Toe walker and foot deformity Foot deformity	Restrictive (subclinical) long QT, transplantation Restrictive Heart Transplantation	Rigid spine multiple contractures Rigid spine Rotatory scoliosis	Subclinical weakness Distal and proximal weakness	Axonal demyelinating neuropathy Axonal neuropathy	No

**Table 2:** LDB3 clinical signs.

Reference	Age Atonset (Years)	Features at Onset	Cardiomyopathy	Contractures	Weakness	Peripheral Neuropathy	Respiratory Failure
Li et al. 2021 [16]	13 years	Gait difficulties	Left ventricular diastolic dysfunction	No	Distal	No	No
Present case	3 years	Gait difficulties	Atrial dilatation, EF 50%	No	Distal	Axonal neuropathy	No

## Data Availability

The datasets generated and/or analyzed during the current study are not publicly available, because they belong to the University of Padova, but are available from the corresponding author (Corrado Angelini) on reasonable request.
